# A copula-infused graph neural network for cell type classification in single-cell RNA sequencing data

**DOI:** 10.1016/j.csbj.2026.01.010

**Published:** 2026-02-02

**Authors:** Shijie Min, Leann Lac, Pingzhao Hu

**Affiliations:** a Dalla Lana School of Public Health, University of Toronto, Toronto, Ontario, Canada; b Department of Computer Science, University of Manitoba, Winnipeg, Manitoba, Canada; c Department of Statistics, University of Manitoba, Winnipeg, Manitoba, Canada; d Department of Computer Science, Western University, London, Ontario, Canada; e Department of Biochemistry, Western University, London, Ontario, Canada

**Keywords:** Single-cell RNA sequencing, Cell type classification, Graph neural networks, Copula theory, ScCopulaGNN

## Abstract

Cell-type classification from single-cell RNA sequencing (scRNA-seq) data is among the most important steps in understanding cellular heterogeneity and biological mechanisms. High dimensionality, sparsity, and noise in scRNA-seq data lead to significant computational and statistical challenges. To this end, we devise a copula-infused graph neural network for single cell type classification (scCopulaGNN). Our model marries the flexibility of copula theory with the strong representation-learning capabilities of graph neural networks. The copula framework naturally captures complex dependencies among genes and the GNN models structural relationships among cells. scCopulaGNN is evaluated on real and simulated datasets and we demonstrate it can handle high-dimensional data with well performance. The model is also compared with existing methods to illustrate the model’s ability to classification task. These results highlight scCopulaGNN potential as an effective tool for cell type classification in single-cell transcriptomics, providing more elaborate details about cellular diversity and function.

## Graphical Abstract

**Figure:**
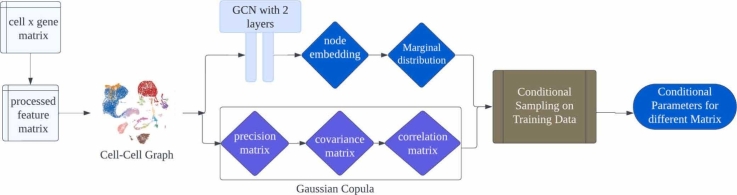

## 1 Introduction

Single-cell RNA sequencing (scRNA-seq) has revolutionized cell biology research by enabling gene expression analysis at the individual cell level, revealing cellular heterogeneity that bulk RNA-seq fails to capture [26]. Unlike traditional sequencing methods, scRNA-seq provides unprecedented insights into cell type diversity and developmental pathways, though it presents significant analytical challenges. The complexity of identifying differentially expressed genes is compounded by cell type diversity, with gene expression variations often attributed to cellular heterogeneity rather than solely genetic regulatory factors [24]. Modern scRNA-seq technology can now process thousands to millions of cells simultaneously, generating intricate data matrices where rows represent individual cells and columns represent genes [21]. Despite its potential, the technique confronts substantial challenges, including high variability, error, and background noise that render traditional statistical modeling inadequate (Jovic et al., 2022). Specialized computational tools have emerged to address these complexities, implementing sophisticated annotation steps to mitigate sequencing biases and missed reads. The bioinformatics workflow remains similar to bulk RNA-seq, encompassing read quality control, mapping, and gene expression estimation [1].

scRNA-seq has evolved from low-sensitivity techniques analyzing few genes [44] to high-throughput technologies capable of quantifying thousands of RNA molecules at single-cell resolution [22], [41]. Platforms like 10x Genomics have dramatically increased throughput and affordability, enabling whole genome and multi-omics analyses that enrich cell biology research [34], [36]. This technology offers unbiased, high-resolution transcriptomic profiling, allowing researchers to characterize precise cell types, explore molecular markers, and identify potential drug targets [27]. By analyzing cells across different time points and disease states, scRNA-seq provides insights into cellular responses, developmental pathways, and critical biological alterations. However, the massive and complex data generated pose significant computational challenges, requiring robust, scalable approaches to address data sparsity, noise, and batch effects [11]. The comprehensive analysis process encompasses data pre-processing, standardization, feature selection, dimensionality reduction, clustering, and differential expression analysis, ultimately empowering researchers to unravel cellular heterogeneity and complex biological systems.

Machine learning methods, particularly clustering algorithms and dimensionality reduction techniques, have become pivotal in cell type prediction and classification for scRNA-seq data [13], [33]. Traditional approaches like K-means and CIBERSORT face limitations in capturing the complexity of biological data, with classifiers such as linear support vector machines and logistic regression showing promise but struggling with accuracy in complex datasets [23]. The inherent challenges of scRNA-seq data, including high variability and class imbalance, necessitate more sophisticated approaches. Recent advancements in deep learning have introduced convolutional neural networks (CNNs) and recurrent neural networks (RNNs) that can process high-dimensional bioinformatics data, revealing intricate biological patterns [25]. Graph neural networks (GNNs) have emerged as a promising solution, offering enhanced modeling of dependencies between nodes and addressing the limitations of traditional deep learning methods [7], [37]. These advanced approaches aim to overcome the shortcomings of earlier techniques by better capturing the complex nonlinear relationships between gene expression and cell types, while accounting for the diversity and heterogeneity inherent in biological data [39]. The evolution of these computational methods represents a critical step in unlocking the full potential of single-cell transcriptomic analysis.

In this paper, we introduce a new innovative technique, specifically coupling the copula method [30], [35], [46] with the strong framework of GNN for scRNA-seq data analysis, which is called scCopulaGNN. Copula theory serves as a sophisticated mathematical tool for modeling complex dependencies among multiple variables. Unlike traditional statistical methods, copulas uniquely separate the marginal distributions of random variables from their joint dependency structure, making them particularly powerful for addressing nonlinear relationships and tail dependencies. Originating from the Latin word "binding", copulas enable the combination of marginal distributions into a comprehensive joint distribution function. This approach allows researchers to capture intricate statistical relationships that conventional linear models might overlook. In the context of single-cell RNA sequencing data analysis, copula theory provides a flexible framework for modeling the complex, often nonlinear interactions between genes and cellular characteristics, thereby offering a more nuanced understanding of cellular heterogeneity and gene expression patterns. In doing this, scCopulaGNN combines GNN strength in dealing with graph-structured data and the flexibility of copula methods to capture complex probabilistic relationships between gene expressions and cell identities effectively. The scCopulaGNN model has been devised to elude the limitations of conventional deep learning methods, gain a more nuanced understanding of the gene expression landscape, and understand its relationship with the different cell types, hence allowing accurate and efficient cell-type prediction.

Simultaneously with GNN grounded models for scRNA seq, modern systems have combined graph structures with transformers to augment long range dependence modeling for cell annotation (e.g., scGraPhT). Probabilistic and adversarial formulations like scVI/scANVI and scNym also tackle batch effects and label transfer at scale. We situate scCopulaGNN as complementary to such work: rather than learn dependencies implicitly with attention or variational prior, we parameterize inter cell dependence explicitly with a copula density linked to GNN derived marginals. This explicit factorization accommodates questioning of dependence structure with strong discriminative features remaining.

## 2 Material and methods

### 2.1 Datasets

#### 2.1.1 Real datasets

In this study, we use four specifically labelled scRNA-seq datasets: Sota, Baron3 [3], Human Kidney [48], and Turtle Brain [42]. Each dataset contains substantial cellular and genetic information and has been labelled for classification purposes. In terms of gene counts, the datasets are comparable, with counts ranging from 20,000 to 24,000, illustrating consistency in gene capture across these studies. Regarding the number of labels, the Sota dataset features 8 labels for 8 cell types, the Baron3 dataset comprises 10 labels, the Human Kidney dataset includes 11 labels, and the Turtle Brain dataset possesses 14 labels. These datasets exhibit significant variations in the number of cells, genes, and labels. The basic statistical characteristics of these datasets are described in Table 1.

**Table 1:** Summary of real labelled scRNA-seq datasets.

**Dataset**	**Description**	**Number of cells**	**Number of genes**	**Number of labels**	**Source**
Sota	Human brain data	420	22085	8	GEO accession number GSM1657871
Baron3	Human pancreas data	3650	20125	10	GEO accession number GSM2230758
Human Kidney	Human kidney congenital mesoblastic nephroma(CMN) data	5685	23797	11	EGA accession number EGAS00001002171
Turtle Brain	Turtle brain data	18664	23500	14	NCBI accession number PRJNA408230

It is essential to apply normalization technique to the raw scRNA-seq data to ensure the gene expression levels are comparable across cells. In our preprocessing, we choose the Centered Log-Ratio (CLR) method. It could transform the gene expression data as a difference in total expression between cells and stabilize the variance across cells. We also reduce the dimensionality using the highly variable genes (HVG) method. This method selects the genes that are most informative and variable. It is important to identify these genes because it could reduce the number of genes or the dimensionality of the data. It will make the cell-type classification task more efficient and prevent there are too many meaningless entries in the data set.

According to biological hypotheses, the cell-cell graph is typically a generalized biological graph facilitating biological functions between cell pairs. Each cell is depicted as a node, and the similarity between two cells is depicted as an edge connecting these nodes. The presence of an edge suggests some similarity between two cells, and the edge weight quantifies this similarity. Specifically, a higher weight denotes greater similarity, while a lower weight indicates lesser similarity [31], [43], [45], [47]. We use the approach to constructing a cell-cell graph from scRNA-seq data using the K-nearest neighbors (K-NN) method. The process involves several steps: initially, gene expression data are preprocessed and normalized; subsequently, pairwise distances are calculated using a suitable metric, such as Euclidean distance. Distance serves as a measure of similarity based on gene expression. Finally, K-NN graph construction is executed, connecting each node to its K-nearest neighbors based on these distances. Edges are established between nodes with the highest similarity, capturing the structural and relational dynamics among cells. Utilizing this structure, the cell-cell graph can elucidate complex cellular relationships and facilitate tasks such as cell type classification, identification of cell subpopulations, and analysis of cellular heterogeneity. For the initial step of the scCopulaGNN, the R package SingleCellExperiment 3.14 is used to construct the K-NN graph from each dataset, with an optimal K of 5 yielding the best results [32].

#### 2.1.2 Simulated datasets

To further evaluate the performance of scCopulaGNN in terms of accuracy for cell label prediction, simulation analysis is conducted using the SPARSim algorithm [4]. According to prior research on scRNA-seq data simulation methods [10], SPARSim has been found highly effective for simulating multiple cell groups, making it appropriate for cell type classification tasks. During the simulation, SPARSim first calculates gene expression level intensities (Z), gene expression level variabilities (Φ), and library size (L) from a selected template to create a dataset. $X_{\mathrm{ij}}$ which is a variable of expression level of gene i in cell j while $Y_{\mathrm{ij}}$ is the read value. In this case, the two variables follow the two distributions

(1) $$X_{\mathrm{ij}}\;∼\;\mathrm{Gamma}(\mathrm{shape}=1/\Phi_{i},\mathrm{scale}=Z_{i}*\Phi_{i}).$$

(2) $$Y_{j}∼\mathrm{Mult}.\mathrm{Hyper}.\left(n=L_{j},m=X_{j}\right).$$

Where $X_{\mathrm{ij}}$ is used to have the biological variability and $Y_{j}$ is to have the technical variability which are the two important factors when creating a scRNA-seq data. In this study, the Zheng dataset is utilized as the labeled template [51].

### 2.2 Proposed model - scCopulaGNN

#### 2.2.1 Introduction to copula

Copula theory serves as a mathematical tool used to depict and model dependency structures among multiple variables. Its primary advantage lies in its ability to distinguish the marginal distributions of multiple random variables from their dependence structure (i.e. joint distribution). This is particularly advantageous when addressing complex nonlinear relationships and tail dependencies. To disaggregate the joint distribution of labels into representational and correlational components, the application of copulas is indispensable. It facilitates the modeling of complex dependencies while preserving the distinct representations of marginal distributions [46]. Originating from the Latin word “binding”, the term “copula” describes a statistical concept that enables the combination of the marginal distributions of multiple random variables into a joint distribution function. According to Sklar’s theorem [38], a copula can link these marginal distributions into a joint distribution, assuming the marginal distributions are continuous. This theorem demonstrates the extensive applicability of copulas in statistical modeling and provides a methodology for examining multivariate relationships by analyzing marginal distributions and dependence structures. It postulates that if *F* is a multivariate joint distribution of a random vector $Y=\left(Y_{1},\ldots .,Y_{n}\right)$ where $F_{i}(y)=P\left(Y_{i}\leq y\right)$ represents the one-dimensional marginal distributions, then *F* can be expressed as

(3) $$F\left(y_{1},\ldots .,y_{n}\right)=C\left(F_{1}\left(y_{1}\right),\ldots ,F_{p}\left(Y_{n}\right)\right)=C\left(u_{1},\ldots .,u_{n}\right).$$

$Y_{i}\;$is the random variable representing gene expression， $y_{i}$ is the realized value or specific observation of $Y_{i}$. In this formula, the joint distribution is viewed as comprising two parts: the marginals and the copulas. Marginals are the $F_{i}$ and the copula is the C. Various types of copulas are available within copula families, such as Gaussian copula, t copula, Gumbel copula. $C:{\left[0,1\right]}^{n}\to [0,1]$ is the copula to describe the dependence structure among those variables. Additionally, a copula C can be regarded as the cumulative distribution function (CDF) of the distribution on ${\left[0,1\right]}^{n}$. Subsequently, the copula density is

(4) $$c\left(u_{1},\ldots .,u_{n}\right)=\partial^{n}C\left(u_{1},\ldots .,u_{n}\right)/\partial u_{1},\ldots .,\partial u_{n}$$

The probability density function (PDF) is represented by the copula density. Assuming the vector Y is continuous, the PDF will be

(5) $$f(y)=c\left(u_{1},\ldots .,u_{n}\right)\prod_{i=1}^{n}\;f_{i}\left(y_{i}\right)$$

where the PDF of $y_{i}$ is $f_{i}$ and c is the copula density.

In practical applications, copula functions are typically determined by selecting a specific copula family, such as Gaussian copula, t copula, or Clayton copula. Each family possesses its own parameters to regulate the strength and type of dependence among variables. By adhering to these parameters, researchers can effectively quantify and model complex dependencies among multiple variables. As numerous copulas exist within the copula family, this paper will focus on one of the most popular ones, the Gaussian copula. A copula is identified as a Gaussian copula if its joint distribution *F* is multivariate normal with a mean of 0 and a covariance matrix of ∑, and the formula is

(6) $$C\left(u_{1},\ldots .,u_{n};\sum \;\right)=\phi_{n}\left(\phi^{-1}\left(u_{1}\right),\ldots .,\phi^{-1}\left(u_{n}\right);0,R\right)$$

where $\phi_{n}\left(\phi^{-1}\left(u_{1}\right),\ldots .,\phi_{n}\left(\phi^{-1}\left(u_{n}\right);0,R\right)\right)$ is the multivariate normal CDF, R is the correlation matrix of ∑, and $\phi^{-1}$ is the quantile function of the univariate standard normal distribution. The copula density for the Gaussian copula is defined as

(7) $$C\left(u_{1},\ldots ,u_{n};\sum \;\right)={\left(\det R\right)}^{-1/2}\exp\left(-\frac{1}{2}\phi^{-1}{\left(u\right)}^{T}\left(R^{-1}-I_{n}\right)\phi^{-1}(u)\right),$$

where $I_{n}$ is an identity matrix and $u=\left(u_{1},\ldots ,u_{n}\right)$. This allows for the modeling of complex multivariate relationships while maintaining the univariate marginal distribution.

In our copula framework, $C\left(u_{1},\ldots .,u_{n}\right)$ represents the multivariate copula function, which captures the dependence structure between random variables by mapping their marginal cumulative distribution functions to a joint distribution. Specifically, $c\left(u_{1},\ldots .,u_{n}\right)$ is the copula density function that describes the local dependence characteristics, where each $u_{i}\in [0,1]$ represents the uniform marginal probability transformed from the original random variables. The significance of $u_{i}$ lies in its ability to standardize different marginal distributions, allowing for a unified representation of dependence across diverse gene expression profiles, independent of the original marginal distributions. This copula approach enables us to model complex interdependencies between gene expressions while preserving the unique characteristics of individual gene distributions.

In analyzing scRNA-seq data, copula theory can be applied to elucidate the dependence structure between genes and to model the similarities between cells. As scRNA-seq data encompasses expression information for thousands of genes, and there exist complex regulatory relationships and functional connections among these genes, it is crucial to employ methods that can encapsulate these complex relationships. Traditional correlation analysis might not fully uncover the nonlinear and tail dependencies among these genes. By offering a flexible modeling framework, copula enables researchers to precisely capture and analyze the dependency structure.

This can be important for various applications including cell clustering and cell type identification. The copula model describes the dependence structure of several dimensions in cell expression profiles. It enables capturing interaction and combinatorial effects between genes. The approach provides a more accurate and all-inclusive way of measuring cellular similarity as compared to the traditional approaches based on single genes or pairwise gene comparison. Relative to the traditional linear model, the copula is more flexible and adaptable in nature, and it can comfortably accommodate various types and strengths of dependencies [35]. Its largest interpretative capability also presents a very versatile tool for modeling with scRNA-seq data analysis, ensuring its perfect use during complex phenomena in the analysis of data.

#### 2.2.2 Graph neural network (GNN)

Unlike conventional neural networks, GNNs capture and model complex relationships among graph nodes, therefore excelling in various knowledge graphs, chemical molecule analyses, and analyses of social networks. What distinguishes GNNs is their high flexibility and expressive capability, allowing them to work on irregular data effectively, with varying structures, and accurately capture complex dependencies between nodes. In practice, GNNs update the node’s representation by aggregating information from the neighboring nodes until reaching a set stopping criterion or maximal number of iterations. The main steps include node feature initialization, messaging, aggregation and update, readout, and prediction. Mainly, GNN conducts many rounds of iteration, in which nodes gather information from their neighbors and constantly update their own features, with the most important one being message passing. The mechanism in the process is an iterative messaging scheme through which GNN can be able to deeply capture complex dependencies in the graph structure, hence demonstrating a powerful expressiveness means in dealing with graph data.

Recently, GNNs have been important to the analysis of scRNA-seq data in general [7], particularly massive single-cell RNA expression datasets from fast-growing repositories such as the Human Cell Atlas. The data obtained by scRNA-seq technology are highly complex, ranging from a large number of relationships between cells to differences in gene expression. Traditional analysis methods result in certain problems because of these complexities. In particular, the great advantages of GNNs in network structure modeling bring new perspectives on scRNA-seq data analysis. By making use of GNNs, one can capture the cell-to-cell interactions effectively, identify clustering patterns of cell types, and reveal the most important gene regulatory networks [17]. The structure-specific abilities of GNNs also allow data dimension reduction, denoising, and feature extraction, thus significantly enhancing the detection of rare cell types and weak signals.

#### 2.2.3 Architecture of scCopulaGNN

The task-specific layer is a critical component of the scCopulaGNN architecture, designed to capture complex features and dependencies in single-cell RNA sequencing data. This layer serves as a sophisticated mechanism for transforming and interpreting graph neural network features. Its architectural design follows a structured approach: receiving input features from the Graph Convolutional Network (GCN), employing a Multi-Layer Perceptron (MLP) for intermediate transformations, and ultimately generating probability distribution predictions. The computational mechanism of this layer is characterized by sophisticated feature processing techniques. Input features undergo non-linear transformations, leveraging advanced neural network architectures to capture intricate relationships between features. To mitigate overfitting, the layer incorporates dropout and regularization techniques, ensuring robust generalization across different datasets. By utilizing multiple neural network layers, the model can effectively extract and represent complex, non-linear interactions between gene expression features. Integration with the graph neural network is achieved through strategic utilization of graph structural information. The layer aggregates features from neighboring nodes, enabling the model to learn sophisticated dependencies between cells. This approach allows the scCopulaGNN to capture the inherent relational structures within single-cell RNA sequencing data, going beyond traditional feature extraction methods. The implementation follows a systematic four-step process: feature extraction, non-linear transformation, probability distribution prediction, and loss function calculation. Each step is carefully designed to enhance the model's ability to classify cell types accurately. By combining copula theory with graph neural network architectures, the task-specific layer provides a powerful mechanism for interpreting the complex, high-dimensional nature of single-cell transcriptomic data.

[30] introduced the CopulaGNN model, which combines Copulas and GNNs to address the challenge of modeling joint distributions in graph-based data which include the graph object G and node feature X. Our proposed model scCopulaGNN has two major innovations based on this method. As the CopulaGNN is used for regression tasks, we adapt our model to handle binary classification tasks, which enhances the ability of scCopulaGNN in cell-type classification tasks. Also, as scCopulaGNN is particularly for scRNA-seq data, it will handle scRNA-seq challenges like high dimensionality and significant cellular heterogeneity. We adapt the highly variable genes (HVG) method for gene selection and generate the cell-cell graph using K-NN method as graph input.

This approach separates the joint distribution of label *y* into two parts: representational and correlational, which may overcome the challenges associated with obtaining the joint distribution. Representational components are reflected in the location parameters $\left(\mu_{i}\mathrm{or}\lambda_{i}\right)$ generated by the base model GCN. The correlational components are depicted in the covariance matrix ∑(X,g,θ) produced by copula. The joint distribution is expressed as:

(8) $$f(y;X,G)=c\left(u_{1},\ldots .,u_{n};X,G\right)\prod_{i=1}^{n}\;f_{i}\left(y_{i};X,G\right),$$

where c is a copula density and $f_{i}$ is a marginal density. In this context, both copula density and marginal densities depend on the graph *G* and the node features ***X***.

For the copulas, the Gaussian copula is selected from the copula family. It is special as it is parameterized by a covariance matrix, where its inverse is the precision matrix. A precision matrix could be correspond directly to a graphical model where zero entries within the matrix could be interpreted as the absence of edges in a graphical model. This characteristic makes Gaussian copula able to map the dependencies within the graph structure, which make it a good choice for our model. On the other hand, other copula families, such as the t-copula or Archimedean copulas, might lack this type of association with graphical models. As Gaussian copula requires the examination of two elements: the covariance matrix and the learning parameter, the relationship between *G* and ∑ in the Gaussian copula density c is considered. Here,$K=\sum^{-1}$ will serve as the precision matrix, and K=0 if the two nodes are not connected. Without restrictions, the number of parameters to be estimated could |ε| and this number might increase.

A potential solution to reduce the number of parameters is to parametrize ***K***. Here, a regression-based parameterization will be employed. This method is notably flexible, as it utilizes a regressor-taking node features to predict non-zero entries in ***K***. Assuming for any node pairs (I,j), a non-zero entry exists in ***K***. Assume $\hat{D}$ is the degree matrix and $\hat{A}$ is the weighted adjacency matrix, we will set ${\hat{A}}_{i,j}=\mathrm{softplus}\left(h\left(x_{i},x_{j};\theta \right)\right)$, where *h* is a two-layer MLP that processes the sequence of $x_{i}$ and $x_{j}$ as input and outputs a scalar. The precision matrix $K=I_{n}+\hat{D}-\hat{A}$. This approach not only makes the estimation of ***K*** more flexible but also maintains the number of learnable parameters *θ* independent of the size *n*. It further ensures that the precision matrix is positive-definite and invertible in calculations.

**Fig. 1:**
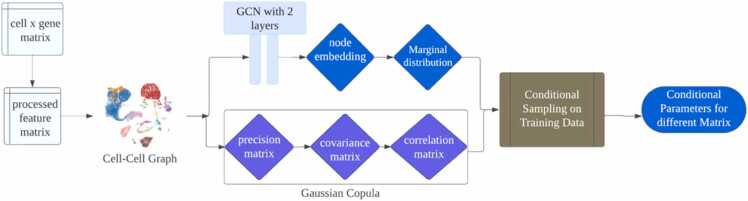
Overview of scCopulaGNN.

One advantage of the copula framework is its flexibility in selecting from different distribution families for modeling marginal densities. When the outcome variable ***y*** is discrete, the choice of Poisson distribution for the marginal densities is deemed appropriate. The marginal density function for the *i*-th outcome is $f_{i}\left(y_{i};\eta_{i}(X,\mathrm{G;}\theta )\right)$, with η(;θ) representing the distribution parameters. If the *i*-th marginal distribution employs a Poisson distribution $\mathrm{Poi}\left(\lambda_{i}\right)$, then $\eta (X,\mathrm{G;}\theta )=\lambda_{i}(X,\mathrm{G;}\theta )$, where $\lambda_{i}(X,\mathrm{G;}\theta )$ is the output of the GCN model for node *i*. Fig. 1 provides a clearer illustration of the scCopulaGNN. The model basically consists of three layers.

**Input layer**: Designed to accept two types of inputs: single-cell RNA sequencing data (***X***) for node characterization and transformed graph data (*G*), which delineates the interrelationships or similarities between cells. In the graph data, each node represents a cell in the scRNA-seq data, accompanied by a preprocessed feature matrix and a node label.

**Hidden layer**: Comprises several GCN layers, each is tailored to decipher complex intercellular interactions and feature representations. Each GCN layer efficiently captures the features of cells and their neighbors through an aggregation function, thereby generating a higher-order representation of the nodes.

**Output layer**: Utilizes the learned feature representations and copula theory to produce a predicted probability distribution for each cell type. This distribution is calculated by integrating the copula density function with the features learned by the GCN.

**Model learning:** One critical parameter in the model that is learned end-to-end is θ, optimized by maximizing the log-likelihood of the observed node labels. This is achieved using the standard optimization algorithm, Adam. As partition of *y* is $y={\left(y_{o\mathrm{bs}}^{T},y_{m\mathrm{iss}}^{T}\right)}^{T}$, the covariance matrix can be partitioned as

(9) $$\left(\begin{array}{cc} \underset{00}{\sum } & \underset{01}{\sum }\\ \underset{10}{\sum } & \underset{11}{\sum } \end{array}\right),$$

where $\sum_{00}\;$ and $\sum_{11}\;$ are the covariance matrices of observed and predicted nodes, respectively.

**Loss function:** A specialized loss function, combining copula and binary cross-entropy loss, is employed. The loss function is formulated as

(10) $$L_{\mathrm{total}}=L(\theta )+L_{\mathrm{BCE}},$$

where L(θ) is copula component and $L_{\mathrm{BCE}}$ is binary cross-entropy loss.

Denoting $u_{\mathrm{obs}}=\left(u_{1},\ldots ,u_{m}\right)$ as the probability integral transform of the label, $y_{i}$ where $\left(u_{\mathrm{miss}}=(u_{\left(m+1\right)}\ldots ,u_{n}\right)$, the copula loss function is given by

(11) $$L(\theta )=-\log f\left(y_{\mathrm{obs}};X,G\right)=-\log c\left(u_{o\mathrm{bs}};\sum \;\right).$$

The binary cross-entropy loss, $L_{\mathrm{BCE}}$ is specified as

(12) $$L_{\mathrm{BCE}}=-1/n\sum_{i=1}^{n}\;\left(y_{i}*\log\hat{y_{i}}+\left(1-y_{i}\right)*\log\left(1-\hat{y_{i}}\right))\right).$$

The choice of an appropriate loss function is dictated by the specific task and output format of our GCN-based framework. Common selections include cross-entropy loss for classification tasks and mean squared error for regression tasks. The loss function measures the discrepancy between the predicted outputs of the model and the ground truth labels or targets, guiding parameter updates during training. By optimizing the chosen loss function, we aim to minimize prediction errors and enhance both the accuracy and performance of our GCN-based framework.

Algorithm 1 Algorithm of scCopulaGNN

**Table:** 

The selection of Gaussian Copula in our scCopulaGNN framework is grounded in its unique mathematical properties and superior performance in modeling complex scRNA-seq data dependencies. Gaussian Copula offers distinctive advantages in capturing non-linear relationships and preserving probabilistic structures inherent in cellular transcriptional networks. Its covariance matrix inverse (precision matrix) provides a direct mapping to graph structural dependencies, enabling precise representation of cellular interactions. Unlike alternative copula families, the Gaussian Copula demonstrates exceptional flexibility in parameter estimation, maintaining computational efficiency while capturing intricate gene expression correlations. Empirical validation across multiple datasets, including Sota, Baron3, Human Kidney, and Turtle Brain, consistently revealed Gaussian Copula's superior performance, with significant improvements in accuracy, ROC-AUC, and precision-recall metrics. The copula's ability to model marginal distributions effectively, particularly when combined with Poisson distributions characteristic of single-cell RNA sequencing data, further substantiates its selection. By leveraging Gaussian Copula's mathematical robustness and adaptability, our model achieves a more nuanced and statistically rigorous approach to single-cell data classification, transcending limitations of traditional linear modeling techniques.

#### 2.2.4 Hyperparameters

Hyperparameters are preset by researchers during model training and are not derived from data learning. Unlike model parameters that are optimized by training data (e.g., weights in a neural network), the selection of hyperparameters directly affects the model's performance, computational time, and generalization ability. Common hyperparameters include learning rate, batch size, number of neural network layers, and number of neurons per layer. The selection of the learning rate is critical for gradient descent algorithms, and an inappropriate learning rate can prolong training time, or cause the model to fail to converge [5]. An experimental study showed that batch size influences not only training efficiency but also generalization ability. In some cases, larger batch sizes may result in the model fitting into a local optimum, while at the same time, smaller batch sizes are shown to enhance the stochastic nature of the model training, thus improving generalization ability. In contrast, the stochastic one is much more effective in very high-dimensional spaces and can thus identify optimum combinations of hyperparameters in a faster fashion compared to traditional grid search [16].

#### 2.2.5 Learning rate and optimization algorithm

The learning rate is an important hyperparameter concerning the size of weight adjustments during parameter updates in each iteration of gradient descent; it governs the speed with which the final value converges. An excessively high learning rate would either overshoot the optimal point or not converge at all, hence failing to train, while a low learning rate brings slow convergence and finally settling into local optima [18]. Empirically, we tune the learning rate such that there is steady progress towards a global optimum without divergence or oscillations.

Then, an optimization algorithm is chosen that best suits the peculiarities of our dataset and model architecture. The most popular choices of optimization algorithm are Adam, an adaptive optimization algorithm that changes learning rates for each parameter, and stochastic gradient descent (SGD), a classical optimization technique that updates parameters based on the average gradient of the loss function over mini batches. We improve the efficiency of training and convergence of the GCN model by selecting appropriate values for learning rates and the optimization algorithm.

### 2.3 Train with unseen data

This ability to generalize would best be tested using unseen data, hence the need for cross-validation. For this reason, it contains the logical flow of information and clear structure with causal links of statements. Cross-validation is a standard technique in model validation, both for statistical and machine learning models, applied when tuning parameters by splitting sample data and repeatedly utilizing the data in training and test sets. Models are first developed on the training set and then tested upon the test set for checking predictive capability. This approach can help researchers use various other models of training and test sets to test the model’s reliability at a more general level. For instance, one training set sample can be in the testing set on another occasion. [2]. This allows the model to be evaluated by all available data, and its suitability for unknown data also has an assessment. Training over unseen data will help prevent overfitting also. The independent validation strategy allows an unbiased estimation of how good the model is in new data because the unseen data offers a surrogate for real-world conditions in which the model could come and the generalization ability be quite imperative if the model is put into practical use (Buterez et al., 2018).

### 2.4 Baseline models

We compare our proposed scCopulaGNN model with six established baseline models which are used as reference to evaluate the proposed model’s performance: Graph Convolutional Network (GCN), Multi-Layer Perceptron (MLP), Graph Attention Network (GAT), GraphSage and two technologies for scRNAseq—Singlecellnet and ACTINN. We chose the deep learning models like GCN, MLP, GAT and GraphSage because they are well-known deep learning models. GCN, GAT and GraphSage are all GNN models that could be used on graph-structured data, which make them good options as baseline models. MLP, as a standard neural network, could provide how our proposed model performs against a more basic deep learning model without graph-based learning. The other two scRNA-seq methods are also important as they could offers more insights about how scCopulaGNN handles the challenges of scRNA-seq data compared with existing methods in this domain.

Graph convolutional network (GCN): GCN is a method within GNN for node classification, which integrates neuron activations to learn representations of graph-structured data. It exploits the graph's structure and node feature information to inform the representation of each node based on its neighborhood [6]. The utility of using GCN to analyze scRNA-seq data is basically attributed to the identification of cell types and the classification of cell subtypes based on the expression pattern and correlation between cells [9], [14], [49], [50]. The GCN baseline model is utilized for comparative purposes regarding performance between the proposed scCopulaGNN model and traditional GNN models, with the help of graph neural networks, in order to look for related explorations of different models in dealing with scRNA-seq data analysis challenges comprehensively in terms of comparison and performance evaluation.

Multi-layer perceptron (MLP): MLP is a classical feed-forward neural network model typically comprising an input layer, several hidden layers, and an output layer [15]. Each layer features multiple neurons linked by weights. When MLP is applied to scRNA-seq data analysis, complex expression patterns and features from a huge number of single-cell data have been extracted, helping in better identification of cell populations that may share gene expression traits. Its strong nonlinear modeling and adaptability suit its preference as a comparative model to test the effectiveness of newly proposed models with applications in cellular data analysis tasks. However, MLPs have the deficiencies of overfitting and sensitivity to parameter selection, which are usually overcome by L1 and L2 regularization techniques along with dropout methods. Also, MLP models are sensitive to the weight initialization and learning rate choices, which in turn affects the stability of the learning process.

Graph attention network (GAT): GAT is a part of GNN and incorporates an attention mechanism for the extraction of complex node-to-node relationships within graph data [28]. In this way, GAT can assign different attention weights to different neighboring nodes around each node, which makes information aggregation more adaptive during message passing, further enhancing the ability of representation learning in graph data. A SoftMax function then normalizes the attention weights. The resulting attention coefficients are used to weigh the features of neighboring nodes to update the representation of each node. With its ability to learn different representations from cell interaction graphs, GAT has turned out to be very efficient for scRNA-seq data analysis and is quite proficient with respect to cell classification tasks [8].

GraphSAGE: is a general inductive graph-based neural network embedding algorithm, generating low-dimensional representations of nodes in very large graphs. The key idea is to learn how to aggregate feature information by considering only a node's local neighbors. GraphSAGE samples a fixed-size subset of instances for aggregating information, instead of using all directly neighboring instances [19]. These features are then aggregated from all the neighboring nodes into the representation of that node. GraphSAGE is an inductive model which can generate embeddings for unseen nodes once the learning is complete.

SingleCellNet: SingleCellNet was developed as a computational tool for classifying cell types and annotating scRNA-seq data. It is based on the machine learning framework that it incorporates to assign a cell type to each one, according to its gene expression profile. The primary goal of SingleCellNet is to be a robust and accurate tool to identify cell types in complex biological samples, which is important in the exploration of cellular diversity and function across different tissues and organisms [12], [20], [40].

Automated cell type identification using neural networks (ACTINN): ACTINN is an approach for cell type identification through scRNA-seq data. It first transforms the scRNA-seq data into a gene pair binary matrix, helping to find out the most informative gene pairs [29]. These important gene pairs are used to train a multi-class random forest classifier. The classifier is trained on these distinguishing features of all cell types and provides high accuracy in predicting cell types from query data. ACTINN performs well across datasets generated from heterogeneous sources and also different sequencing technologies, making it reliable for cell type identification.

Other significant baselines In order to remain competitive with modern single cell annotation systems, we also take into account two families of new state of the art (SOTA) approaches in addition to standard GNN/MLP baselines: (i) graph–transformer hybrids like scGraPhT, which combines transformer attention with graph message passing at the task of cell type annotation; and (ii) broadly used scRNA seq classifiers like scVI/scANVI or scNym/Celltypist, which represent single cell count data with probabilistic or adversarial objectives designed to address batch effects and label transfer. Wherein lies an official implementation at a public venue, we assess the approach with the authors’ bench mark code and authors’recommended preprocessing; otherwise we document the approach and provide a methodological comparison in place of a head to head run. This procedure forestalls inadvertent biases due to re-implementations and is consistent with the community standard for fair benchmarking.

Graph–transformer hybrids are configured to learn long range transcriptomic dependencies along with cell–cell topology at the same time, whereas scVI family and corresponding classifiers have good label transfer under batch effects baselines. Adding both families supplements our GNN focused baselines ((GCN, GraphSAGE, GAT) and scRNA seq specificutils (SingleCellNet, ACTINN) to form a balanced view across modeling paradigm

### 2.5 Model evaluation

In our scCopulaGNN study, we implemented a rigorous 5-fold cross-validation methodology to ensure robust model performance and generalizability in single-cell RNA sequencing analysis. By systematically dividing the dataset into five equally sized subsets and iteratively using four subsets for training and one for validation, we comprehensively assessed the model's predictive capabilities. This approach allowed us to test the model's ability to generalize to unseen data, mitigate overfitting risks, and provide a reliable performance evaluation across diverse cellular datasets. We calculated key performance metrics including accuracy, precision, recall, F1-score, and ROC-AUC for each fold, ultimately computing average performance metrics. The 5-fold cross-validation strategy balanced computational efficiency with comprehensive model evaluation, generating unbiased performance estimates and simulating real-world application scenarios, thereby demonstrating the scCopulaGNN's potential for broader scientific applications in single-cell data analysis.

## 3 Results

### 3.1 Selection of parameters

#### 3.1.1 Choice of number of genes and K

The proposed method was implemented through highly variable genes (HVG), a widely used filtering method in genes to get genes expressed with high dispersion across various samples. Such a selection procedure ensures the identification of highly informative genes, aiding in the process of distinguishing between various conditions or cell types. We use Human Kidney dataset here to perform the analysis. As described in Table 2, while the model involving 2000 genes was the best on average - average accuracy: 0.7080, the 1200-gene model achieved overall better performance in terms of balanced metrics, with an ROC-AUC of 0.9704 and PR-AUC of 0.9174 compared to 0.9665 and 0.9077 for 2000 genes, respectively. Averaging with less ROC-AUC and PR-AUC was shown in the model with 1000 genes, meaning worse performance. Hence, the 1200 genes were chosen because they gave an improved balance of accuracy with correct classification between positive and negative instances, and therefore proved to be the fittest for our analysis.

**Table 2:** Model performance of Different Number of Gene on Human Kidney dataset.

**Number of genes**	**Average accuracy**	**Average ROC-AUC**	**Average PR-AUC**
1200	0.6186	0.9704	0.9174
1000	0.6331	0.9590	0.8387
2000	0.7080	0.9665	0.9077

In this study, after a detailed performance analysis conducted based on average accuracy, average ROC-AUC, and average PR-AUC, K= 5 was found to be the best parameter value for K in our model with the use of 1200 genes (Table 3). Although K= 10 and K= 15 gave higher values for the ROC-AUC (0.9788 compared to 0.9704 for K=5), an average accuracy of 0.6186 and PR-AUC of 0.9174, respectively, favored K= 5. According to all these metrics, even if K= 10 and K= 15 perform a little better in class discrimination, still K= 5 has a more balanced and robust performance based on all the other metrics. This balance is crucial for our model as it ensures high accuracy and reliability in predicting true positive and true negative cases. Therefore, K= 5 was chosen to maintain a strong overall performance and to leverage the comprehensive benefits of using 1200 genes identified through the HVG method.

**Table 3:** Model Performance of Different Number of K on Human Kidney dataset.

**Num of K**	**Average accuracy**	**Average ROC-AUC**	**Average PR-AUC**
K= 5	0.6186	0.9704	0.9174
K= 10	0.6153	0.9788	0.8712
K= 15	0.6109	0.9788	0.8656

#### 3.1.2 Choice of marginal distribution

In this study, the Poisson distribution was used to model the marginal densities within the copula framework. When outcome variables are discrete, as in our case, the Poisson distribution is particularly appropriate because it effectively models count data, which aligns with the nature of our outcomes. It was chosen based on superior model performance metrics in Human Kidney dataset, where the Poisson distribution achieved significantly higher average accuracy (0.6186), average ROC-AUC (0.9704), and average PR-AUC (0.9174) compared to the binomial distribution. The performance of the binomial distribution, with much lower metrics, underscores the appropriateness of using the Poisson distribution for discrete outcome variables in our copula framework, as described in Table 4.

**Table 4:** Model Performance of different marginal distribution on Human Kidney dataset.

**Marginal distribution**	**Average accuracy**	**Average ROC-AUC**	**Average PR-AUC**
Poisson	0.6186	0.9704	0.9174
Binomial	0.0764	0.3818	0.0789

### 3.2 Ablation analysis

Ablation analysis was conducted to evaluate the distinct contributions of the copula and GNN components within the scCopulaGNN framework.

#### 3.2.1 Human Kidney dataset

The results, as shown in Table 5, provide clear insights into the impact of each model on overall performance on Human Kidney dataset. The scCopulaGNN model demonstrated the highest performance across all metrics, with an average accuracy of 0.6186, an average ROC-AUC of 0.9704, and an average PR-AUC of 0.9174. This highlights the effectiveness of integrating both copula and GNN components, where the GNN captures the feature representations of nodes, and the copula models the dependencies among nodes. The copula model was the model that demonstrated worse performance hardly, achieving robust predictive performance: an average accuracy of 0.4533, an average ROC-AUC of 0.5000, and an average PR-AUC of 0.5382. This means that while it can model dependencies effectively through the copula, it is unable to model the fine-grained features that are key to making accurate predictions from the graph data. On the other hand, performance on the GCN model, addressing exclusively the learning of node features and their direct interactions but not modeling dependencies through copulas was quite good. The average accuracy for this model was 0.4921, and the average ROC-AUC and PR-AUC were 0.9968 and 0.9860, respectively. The high ROC-AUC and PR-AUC values reveal that the GNN is quite good in class separation. However, an equally slightly lower average accuracy indicates that there might be some problems with overall predictive performance. This is most likely due to the fact that while a GNN is able to learn feature representations, it is constrained in the learning of complex dependencies between nodes that the copula component handles.

**Table 5:** Ablation analysis of model performance on Human Kidney dataset.

**Model**	**Average accuracy**	**Average ROC-AUC**	**Average PR-AUC**
scCopulaGNN	0.6186	0.9704	0.9174
Copula	0.4533	0.5000	0.5382
GCN	0.4921	0.9968	0.9860

#### 3.2.2 Baron3 dataset

We also conducted an ablation analysis on the Baron3 dataset to assess the individual contributions of the copula and GNN components within the scCopulaGNN framework, with the results presented in Table 6.

**Table 6:** Ablation analysis of model performance on Baron3 dataset.

**Model**	**Average Accuracy**	**Average ROC-AUC**	**Average PR-AUC**
scCopulaGNN	0.6102	0.9953	0.9803
Copula	0.4831	0.5000	0.5503
GCN	0.6861	0.9942	0.9815

Overall, the scCopulaGNN model performed well in all evaluation metrics, with an average accuracy of 0.6102, an average ROC-AUC of 0.9953, and an average PR-AUC of 0.9803, which reflects the combined efficacy of both copula and GNN components. The copula component will model the dependencies among such nodes in a way that the end predictive model is reliable and robust since the GNN model is well set to capture the feature representations of nodes. The copula model, with its independence on the modeling of dependencies between the nodes, was comparably less efficient. This model had poor predictive power, having an average accuracy of 0.4831, average ROC-AUC of 0.5000, and average PR-AUC of 0.5503. This, in fact, means that while the copula component can capture the structure of dependence quite well, it simply does not have the ability to learn fine-grained representations of features from graph data, which is a key point for accurate predictions of cell type. On the other hand, the GCN model is focused on learning the features of nodes and their direct interactions, independent of the copula framework in modeling dependencies, with an average accuracy of 0.6861. A high value is also held in respect to the average ROC-AUC (0.9942) and PR-AUC (0.9815). Therefore, from the high ROC-AUC and PR-AUC, it can be seen that the GCN may be able to do very well at distinguishing between classes. However, its somehow lower accuracy compared to the scCopulaGNN model clearly points to potential limitations regarding the overall predictive performance.

As we see a very similar result of the ablation analysis as observed with the Human Kidney dataset, it indicates a good support from the copula and GNN components of the scCopulaGNN model. The copula framework models dependencies and correlations among the nodes quite well, while the GNN component learns good feature representations from the nodes. The performance of the scCopulaGNN model is much more balanced across showing better accuracy and reliable classification metrics. This is underlined in the analysis, but only the integration of both components can lead to the best performance in predicting cell type.

### 3.3 Analysis of real-world datasets

We apply the proposed method to real-world datasets and calculate accuracy, ROC-AUC, and PR-AUC values in comparison to the baseline models.

#### 3.3.1 Sota dataset

The Sota dataset is the smallest real-world dataset, containing only 420 cells and 8 different cell types. The results in Table 7 from the reported performances among a variety of models on the Sota dataset illustrate the impressively strong ability of the scCopulaGNN model. On average, the scCopulaGNN model was able to achieve 0.9911 accuracy, which is indeed the best in comparison to the other alternative models and hence can classify cell types in the dataset correctly. In average ROC-AUC, scCopulaGNN reached 0.9960, meaning that in general, it is really strong in discriminating positive and negative classes. While GCN, SAGE, and ACTINN reached a little higher ROC-AUC of 0.9991, 0.9993, and 0.9991, respectively, the results of scCopulaGNN are still high.

**Table 7:** Performance of scCopulaGNN and baseline models on Sota dataset.

**Model**	**Average Accuracy**	**Average ROC-AUC**	**Average PR-AUC**
scCopulaGNN	0.9911	0.9960	0.9981
GAT	0.9286	0.9983	0.9864
GCN	0.9196	0.9991	0.9961
MLP	0.8929	0.9978	0.9725
SAGE	0.9315	0.9993	0.9985
SingleCellNet	0.9414	0.9975	0.9755
ACTINN	0.9880	0.9991	0.9424

**Fig. 2:**
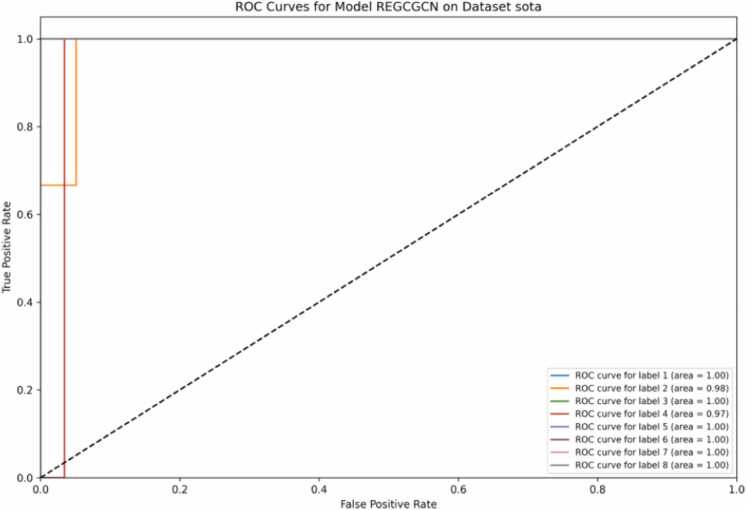
ROC curves of scCopulaGNN on Sota dataset.

As for the average PR-AUC metric, the scCopulaGNN model showed the best performance. An outstanding result of 0.9981 among all means that the scCopulaGNN could balance effectively between precision and recall, demonstrating good handling of imbalanced datasets. Other kinds of models are SAGE, with a PR-AUC of 0.9985, and GCN, with 0.9961. From Fig. 2, the scCopulaGNN model obtains ideal AUC values of 1.00 for labels 1, 3, 5, 6, 7, and 8, which means really good capability to classify these labels correctly. These moderate-sized labels can be handled by the model, although there will generally be some fluctuation of accuracy in the results. For instance, label 1 is classified perfectly and has 110 samples, indicating the capability of the model in handling medium-sized labels. Similarly, label 4, the largest with 131 samples, has an AUC of 0.97, reflecting the model's robustness even with larger label sizes.

Less-represented labels, like label 2 with 25 samples and label 5 with only 20 samples, also perform at high levels: AUC values of 0.98 and 1.00, respectively. This shows the great ability of the model to separate the classes well even in cases with a lesser number of samples.

#### 3.3.2 Baron3 dataset

**Fig. 3:**
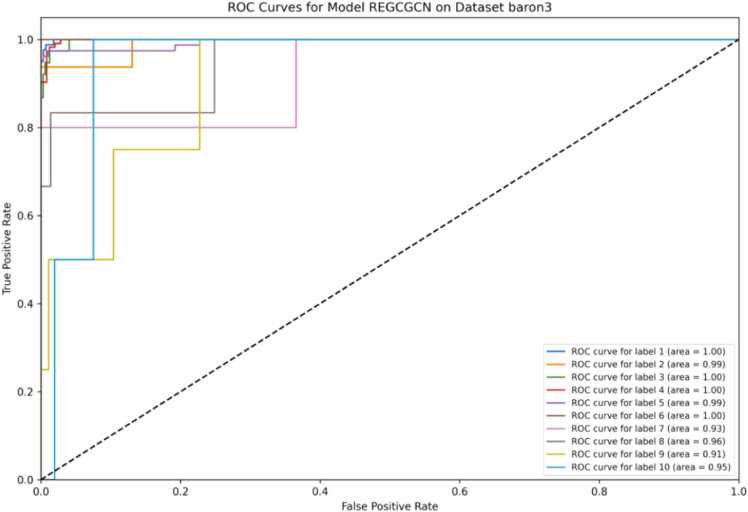
ROC Curves of scCopulaGNN for Baron3 dataset.

The third important dataset on which the cell-type classification task is performed is the Baron3 dataset. As described in Fig. 3 and Table 8, the ROC curve for the model scCopulaGNN on the baron3 dataset shows great performance on several labels witnessed by AUC values between 0.91 and 1.00.

**Table 8:** Performance of scCopulaGNN and baseline models on Baron3 dataset.

**Model**	**Average accuracy**	**Average ROC-AUC**	**Average PR-AUC**
scCopulaGNN	0.6102	0.9953	0.9803
GAT	0.6093	0.9867	0.9738
GCN	0.6861	0.9942	0.9815
MLP	0.6491	0.9881	0.9799
SAGE	0.7009	0.9909	0.9803
SingleCellNet	0.6435	0.9866	0.9756
ACTINN	0.6655	0.9889	0.9804

The model achieves perfect AUC values of 1.00 for labels 1, 3, 4, and 6, regardless of the varying sizes of these labels. For example, label 4, with the largest sample size of 1130, still performs perfectly. Similarly, labels with smaller sample sizes, such as label 6 with 92 samples, also achieve an AUC of 1.00, indicating that the model is uniformly effective across different sample sizes. Labels 2 and 5, with AUC values of 0.99, also have relatively high sample sizes (161 and 787), which may illustrate the model’s consistency in classifying cell types. The performance for labels 7, 9, and 10, although with slightly lower ROC-AUC values of 0.93, 0.91, and 0.95, still reflects strong predictive capability as all these values exceed 0.9, demonstrating that the model is effective at classifying cell types.

The ROC curves combined with the label size data support the conclusion that the scCopulaGNN model is highly effective across a diverse range of label sizes within the Baron3 dataset. The high AUC values confirm that the model maintains excellent classification accuracy, even with imbalanced label distributions. Detailed performance results of scCopulaGNN in comparison to baselines for Baron3 dataset in classifying label 1 is provided in Supplementary Section 1, Figures S1, S2, S3, and S4.

#### 3.3.3 Human Kidney dataset

The Human Kidney dataset provides a comprehensive account of scRNA-seq data from human kidney cells, which describes complex heterogeneities in cell expression occurring in the human kidney.

The results show that scCopulaGNN successfully outperformed other models (Table 9), achieving an accuracy of 0.6186, which is far better than the rest. This suggests that scCopulaGNN classifies cell types for label 1 very well. SAGE also performed great with an accuracy of 0.5272. The relatively low level of accuracy for GAT and the others argues about their demerits as compared to the two above-mentioned models. The best AUC for the ROC curve exhibited under the performance of the SAGE model is 0.9976, showing its potential to separate among different cell types. GCN and MLP also showed strong performance, with AUC values of 0.9968 and 0.9966, respectively. The scCopulaGNN model, with an AUC of 0.9704, though slightly lower than these, still displays considerable discriminatory power. The SAGE model achieved the highest PR-AUC of 0.9894, followed closely by GCN and MLP. The scCopulaGNN model’s PR-AUC of 0.9174, while lower compared to other models, still remains impressive as it exceeds 0.9, indicating its proficiency in handling imbalanced datasets. Detailed performance results of scCopulaGNN in comparison to baselines for Human Kidney dataset in classifying label 1 is provided in Supplementary Section 2, Figures S5, S6, S7, and S8.

**Table 9:** Model Performance of Human Kidney.

**Model**	**Average Accuracy**	**Average ROC-AUC**	**Average PR-AUC**
scCopulaGNN	0.6186	0.9704	0.9174
GAT	0.3286	0.9928	0.9748
GCN	0.4921	0.9968	0.9860
MLP	0.4455	0.9966	0.9856
SAGE	0.5272	0.9976	0.9894
SingleCellNet	0.4691	0.9858	0.9698
ACTINN	0.4507	0.9904	0.9399

#### 3.3.4 Turtle brain dataset

Finally, we also include here one unrelated dataset, Turtle Brain dataset. It actually contains the highest cell number in this study: 18,664. As we can see from Table 10, scCopulaGNN has the best average accuracy of 0.9932 and continues to show outstanding performance in obtaining an accurate classification of cell types within a large dataset. Despite its impressive accuracy, scCopulaGNN’s average ROC-AUC is 0.8720, which is superior when compared to other models, highlighting its capability to distinguish between positive and negative classes. The average PR-AUC for scCopulaGNN is 0.4808, which is not particularly high. However, other baseline models exhibit very low PR-AUC values, suggesting their inability to effectively manage large datasets with imbalance data. High accuracy with low PR-AUC indicates a class-imbalance artifact: most predictions are on majority labels. PR-AUC is thus the more decision-relevant metric here.

**Table 10:** Performance of scCopulaGNN and baselines on Turtle Brain dataset.

**Model**	**Average accuracy**	**Average ROC-AUC**	**Average PR-AUC**
scCopulaGNN	0.9932	0.8720	0.4808
GAT	0.9333	0.6055	0.1974
GCN	0.9333	0.5747	0.1460
MLP	0.9333	0.5946	0.1802
SAGE	0.9321	0.6062	0.2021
SingleCellNet	0.8986	0.5600	0.2327
ACTINN	0.9369	0.7882	0.5158

Other models, such as GAT, GCN, and MLP, all with an average accuracy of 0.9333, display significantly lower average ROC-AUC values of 0.6055, 0.5747, and 0.5946, respectively. Their average PR-AUC values, ranging from 0.1460 to 0.1974, further highlight their challenges in the precision-recall trade-offs within this large dataset. SAGE, with an average accuracy of 0.9321, also struggles with this issue, having an ROC-AUC of 0.6062 and a PR-AUC of 0.2021. ACTINN, while achieving a high average accuracy of 0.9369 and a ROC-AUC of 0.7882, has a PR-AUC of 0.5158, which is better than that of scCopulaGNN. However, scCopulaGNN’s overall performance, particularly in terms of accuracy, illustrates its capability to classify cell types in large-scale datasets.

### 3.4 Simulated datasets

#### 3.4.1 Z-mid dataset

Here, we first analyze the Z-mid dataset, which comprises 6776 cells across 4 labels. The performance of various models on simulated data is summarized in Table 11. Our proposed model, scCopulaGNN, outperforms all other models across the evaluated metrics, achieving an average accuracy of 0.9703, an average ROC-AUC of 0.9684, and an average PR-AUC of 0.8573. These highlights scCopulaGNN's strong classification capabilities. The ROC-AUC value further demonstrates its ability to distinguish between the labels, a strength also reflected in its PR-AUC. In contrast, the GAT model exhibits significantly lower performance, with an average accuracy of 0.6830, an average ROC-AUC of 0.7322, and an average PR-AUC of 0.7729. Other models, including GCN, MLP, SAGE, SingleCellNet, and ACTINN, display varying performances, but none approach the overall effectiveness of scCopulaGNN. Notably, models such as GCN, MLP, and SAGE achieve high PR-AUC scores of 0.9754, 0.9749, and 0.9759, suggesting a strong balance of precision and recall, yet their overall accuracy and ROC-AUC scores are lower than those of scCopulaGNN. These results underscore scCopulaGNN's ability to accurately classify cell types in simulated data. More details regarding performance results of scCopulaGNN in comparison to baselines for simulated dataset Z-mid in classifying label 1 is provided in Supplementary Section 3, Figures S8, S9, S10, and S11.

**Table 11:** Performance of scCopulaGNN and baselines on simulated data (Z-mid).

**Model**	**Average Accuracy**	**Average ROC-AUC**	**Average PR-AUC**
scCopulaGNN	0.9703	0.9684	0.8573
GAT	0.6830	0.7322	0.7729
GCN	0.8298	0.8377	0.9754
MLP	0.8333	0.8424	0.9749
SAGE	0.8308	0.8390	0.9759
SingleCellNet	0.8382	0.8650	0.8380
ACTINN	0.7820	0.7874	0.8219

#### 3.4.2 Z-half dataset

The Z-half dataset is a simulated dataset created using the same method as the Z-mid dataset. It comprises 1694 cells across four labels, making it a relatively small dataset compared to the Z-mid dataset. The high average performance of the scCopulaGNN model was 0.9174 accuracy, an outstanding average ROC-AUC of 0.9979, and a high average PR-AUC of 0.9441 (Table 12). It speaks to the high effectiveness of scCopulaGNN in the accurate classification and differentiation between many labels, hence making it a model suitable for the dataset. These results underscore how dependable the model is for similar datasets.

**Table 12:** Performance of scCopulaGNN and baselines on simulated data (Z-half).

**Model**	**Average Accuracy**	**Average ROC-AUC**	**Average PR-AUC**
scCopulaGNN	0.9174	0.9979	0.9441
GAT	0.8948	0.8163	0.9823
GCN	0.9830	0.8750	0.8820
MLP	0.9794	0.7470	0.7559
SAGE	0.9794	0.7470	0.7559
SingleCellNet	0.7294	0.5000	0.6353
ACTINN	0.9897	0.9590	0.8702

The GAT model showed the least average accuracy among models, with a mean of 0.8948 and an average ROC-AUC of 0.8163. In return, it shined in PR-AUC at a high level of 0.9823. The GCN model showed the best average accuracy of 0.9830, with the average ROC-AUC at 0.8750 and the average PR-AUC at 0.8820, which suggests that while GCN is very good in classifying classes, scCopulaGNN is better at doing this. Two other models, MLP and SAGE, show the highest accuracy, with an average of 0.9794; their ROC-AUC and PR-AUC fall within scores of 0.7470 and 0.7559. Thus, they performed similar to most of the models among the collection, but the discrimination ability is not as strong as for some others. SingleCellNet underperformed in all criteria: accuracy at 0.7294, ROC-AUC at 0.5000, and PR-AUC at 0.6353. This testifies to the great and significant difficulties in the way of an accurate classification when the model separates objects into different classes. Whereas ACTINN, being at the top in terms of performance average, reaches 0.9897 for accuracy, 0.9590 for ROC-AUC and average PR-AUC of 0.8702 robust. In spite of the proven performance by ACTINN, scCopulaGNN also holds its place at the top.

#### 3.4.3 Z-same dataset

Here we benchmark all models on an extended simulated dataset of 3388 cells and 4 labels. The benchmarked models include scCopulaGNN, GAT, GCN, MLP, SAGE, SingleCellNet, and ACTINN. We benchmark in terms of three primary metrics that include average accuracy, average ROC-AUC, and average PR-AUC.

The average accuracy in the test dataset was quite high, which is 0.9853, along with a high average ROC-AUC of 0.9800 and PR-AUC of 0.8435, respectively (Table 13). These results confirm that scCopulaGNN continues to perform robustly for accurate classification of cells and distinguishing between different labels, even for increased dataset size.

**Table 13:** Performance of scCopulaGNN and baselines on simulated data (Z-same).

**Model**	**Average Accuracy**	**Average ROC-AUC**	**Average PR-AUC**
scCopulaGNN	0.9853	0.9800	0.8435
GAT	0.7493	0.5000	0.6254
GCN	0.9742	0.7494	0.7580
MLP	0.9830	0.8750	0.8820
SAGE	0.9808	0.7500	0.7588
SingleCellNet	0.8947	0.8163	0.9823
ACTINN	0.7500	1.0000	0.9997

In summary, on the other hand, the average accuracy was at 0.7493, the average ROC-AUC was 0.5000, and the average PR-AUC was 0.6254 in the general case. Such metrics, on one hand, show that GAT does not work well over a larger dataset in differentiating classes, which is pretty evident due to its lower ROC-AUC. An average accuracy of 0.9742, an ROC-AUC of 0.7494, and a PR-AUC of 0.7580 are recorded in the GCN model. Although GCN attains high accuracy, its ROC-AUC and PR-AUC are much lower than those of scCopulaGNN, indicating less effective performance in cell-type classification. The MLP model gave great results with an average accuracy of 0.9830, ROC-AUC of 0.8750, and PR-AUC of 0.8820 but did not perform as well in ROC-AUC as the scCopulaGNN model.

On average, SAGE had an accuracy of 0.9808, with an ROC-AUC of 0.7500 and a PR-AUC of 0.7588. From these statistics, one can deduce that SAGE, while performing on average like GCN, is being outperformed by scCopulaGNN in label classification. SingleCellNet obtained an average accuracy of 0.8947, whereas both the ROC-AUC and PR-AUC were 0.8163. However, in general performance, although the PR-AUC was very high, it fell just short of scCopulaGNN, especially by accuracy and ROC-AUC. The average accuracy for the ACTINN model is 0.7500, the value of ROC-AUC is 1.0000, and the average PR-AUC is 0.9997. Even though there is perfect class separation, its slightly lower accuracy might reflect problems with the correct classification of a higher ratio of cells compared to scCopulaGNN. We have collected performance results of scCopulaGNN in comparison to baselines for simulated dataset Z-same in classifying label 1. Detailed results are provided in Supplementary Section 4, Figures S12.

### 3.5 Effect of different label size

We measured performance at label size strata levels in order to see what happens under class imbalance. Labels for each data set were sorted by sample frequency and divided into quintiles; statistics were aggregated within strata. Since precision–recall is a more informative measure than ROC under imbalance, we present macroaveraged PR AUC as the lead metric, with accuracy and ROC AUC given as background.

From this analysis, it can be deduced that generally, bigger label sizes produce better average accuracy. For instance, labels 1 and 7 with 1201 and 1498 observations, respectively achieved the best average accuracies of 0.7364 and 0.7645, respectively. This means that with more labels comes more information and knowledge, meaning the model improves its learning ability as well as performance. The enhanced data helped the model recognize underlying patterns more accurately, hence improving prediction accuracy. In ROC-AUC, the model has performed the best on the test with label 2, where 268 observations were made, label 6 with 259, and label 7 with 1498 observations. This means that the model is significantly better in differentiating between classes with respect to moderate-sized labels. On the other hand, the performance of the ROC-AUC is relatively lower for smaller labels such as label 4 with 60 observations and label 8 with 118 observations, performing at 0.9136 and 0.9283, respectively. This reduction might be due to lack of data that would help the model effectively discriminate between classes.

The PR-AUC values also point to a positive correlation towards larger label sizes. Labels 2, 6, and 7 had the highest PR-AUC scores at 0.9958, 0.9985, and 0.9934, respectively. Increased label size aids the model in its support of identifying true positives with a low false positive rate. In contrast, label 8, with a mere 118 observations, marked a markedly lower PR-AUC of 0.4863, showing explicitly the trouble of attaining precision and recall with fewer labels. In some cases, the model maintains relatively high performance despite smaller counts in the labels. For example, label size 3 with 731 observations has an average accuracy of 0.7118, average ROC-AUC of 0.9796, and an average PR-AUC of 0.9509. This means that though larger label sizes assist a model to perform better overall, the model can still be pretty decent with moderate label sizes. The analysis again confirms that a larger size of the labels generally boosts the performance of the scCopulaGNN model but still remains effective within a good range of sizes. The design of the model allows it to perform adequately even if lesser labels are available, thereby proving its adaptability in the face of varying amounts of labeled data.

To assess the impact of label size on the scCopulaGNN model's performance, we further evaluated the model's metrics across varying label sizes in the Baron3 dataset, as detailed in Table 14, Table 15. This analysis clarifies the influence of label quantity on the model's accuracy, ROC-AUC, and PR-AUC for this dataset.

**Table 14:** Performance of scCopulaGNN on Human Kidney dataset for different labels.

**Label**	**Label size**	**Average accuracy**	**Average ROC-AUC**	**Average PR-AUC**
1	1201	0.7364	0.9682	0.9002
2	268	0.6924	0.9998	0.9958
3	731	0.7118	0.9796	0.9509
4	60	0.6678	0.9136	0.8364
5	373	0.6503	0.9488	0.8524
6	259	0.7047	0.9999	0.9985
7	1498	0.7645	0.9974	0.9934
8	118	0.6889	0.9283	0.4863
9	73	0.6503	0.9655	0.7027
10	483	0.6960	0.9587	0.8881
11	621	0.6907	0.9989	0.9930

**Table 15:** Model Performance of Different Label Size with dataset Baron3.

**Label**	**Label Size**	**Average Accuracy**	**Average ROC-AUC**	**Average PR-AUC**
1	843	0.6889	0.9999	0.9997
2	161	0.7028	0.9871	0.9487
3	376	0.7278	0.9998	0.9986
4	1130	0.8361	0.9998	0.9995
5	787	0.8333	0.9942	0.9895
6	92	0.6889	1.0000	1.0000
7	100	0.7722	0.9483	0.8065
8	54	0.7389	0.9614	0.7925
9	36	0.7417	0.9607	0.4119
10	14	0.7389	0.9888	0.5778

The results illustrated that similarly to the Human Kidney dataset, larger sizes of labels in the Baron3 dataset generally contribute to higher average accuracy. For instance, label 4, the largest one, with 1130 cells and label 5, with 787 cells, have led to the two most successful average accuracies of 0.8361 and 0.8333, respectively. This would mean that more labels lead to better learning in the model, meaning the understanding of the patterns is proper, and hence the model gives better performance because it becomes more accurate.

The model shows quite a brilliant performance on the ROC-AUC for different sizes of the labels. Each of the labels, 1, 3, and 6, reached the ROC-AUC value close to 1, which indicates robustness in the ability of the model to distinguish between classes. Even smaller sizes of the label, like the label 10, are holding their ROC-AUC high at 0.9888, showing great effectiveness of the model in the ability of distinguishing classes with little data.

The PR-AUC metrics also indicate improved performance with larger label sizes, paralleling results from the Human Kidney dataset. Labels 1, 3, and 4 achieved the highest PR-AUC values of 0.9997, 0.9986, and 0.9995, respectively. This shows that larger label sizes facilitate the model's ability to accurately identify true positives while reducing false positives. Interestingly, the model also maintains commendable performance with smaller label sizes in certain instances. For example, label 7 achieved an average accuracy of 0.7722, an average ROC-AUC of 0.9483, and an average PR-AUC of 0.8065. This demonstrates that while larger label sizes can enhance performance, the scCopulaGNN model remains capable of performing well with moderate label sizes.

The analysis confirms that in the Baron3 dataset, as observed with the Human Kidney dataset, larger label sizes generally boost the scCopulaGNN model’s performance. The increased data volume affords more learning opportunities for the model, leading to higher accuracy and superior classification metrics.

### 3.6 Train with unseen data

We also evaluate model performance on unseen data. For this purpose, we utilize the Human PBMC dataset to train the models. As demonstrated in Table 16, scCopulaGNN achieves a balanced performance with an average accuracy of 0.7692, ROC-AUC of 0.5405, and PR-AUC of 0.5341, showcasing its moderate ability to learn and perform on new data. This performance is particularly notable when compared to other models such as GAT, GCN, MLP, SAGE, SingleCellNet, and ACTINN, each displaying unique strengths and weaknesses.

**Table 16:** Performance of scCopulaGNN and baselines on unseen data.

**Model**	**Average accuracy**	**Average ROC-AUC**	**Average PR-AUC**
scCopulaGNN	0.7692	0.5405	0.5341
GAT	0.5988	0.4890	0.3077
GCN	0.7233	0.5723	0.4254
MLP	0.6875	0.5468	0.4695
SAGE	0.7142	0.5190	0.4362
SingleCellNet	0.4388	0.9455	0.9889
ACTINN	0.8576	0.4158	0.1266

In a comparative analysis, scCopulaGNN outperforms models like GAT, which shows lower average accuracy (0.5988), ROC-AUC (0.4890), and PR-AUC (0.3077), underscoring scCopulaGNN's superior efficiency in processing unseen data. Conversely, GCN, with an average accuracy of 0.7233 and ROC-AUC of 0.5723, demonstrates a slightly better ability to distinguish between classes compared to scCopulaGNN, but records a lower PR-AUC (0.4254), indicating that scCopulaGNN provides a more consistent performance across various metrics. SingleCellNet achieved a high ROC-AUC of 0.9455 and PR-AUC of 0.9889, but much lower average accuracy of 0.4388, indicating it to be quite capable of picking up true positive signatures while failing at general prediction accuracy. On the contrary, ACTINN displayed the highest average accuracy of 0.8576, alongside lower ROC-AUC (0.4158) and PR-AUC (0.1266), possibly showing signs of struggle in porting this model with new datasets.

Heterogeneous behaviors across datasets are found in prelminary results: scCopulaGNN competes on moderate to large labels and potentially wins on very small labels on certain datasets, while traditional classifiers are advantageuos on others. Under these dataset specific behaviors, we are self-conscious about making universal arguments that small label dominance occurs. Rather, we point out that the copula term can calibrate decisions by linking sparse labels to the overall dependence structure, which is nicest whenever local evidence ((marginals) is not strong.

## 4 Discussion

### 4.1 Performance in simulated data

The scCopulaGNN model showed superior performance over the simulated datasets. We simulated the datasets with full prior knowledge: that is, scCopulaGNN performance was tested based on scenarios for other single-cell tissues. In the simulated datasets, scCopulaGNN performed accurately, which attested to its effectiveness in the correct classification of cell types. This was largely due to the ability to handle the huge number of features, as well as the complicated interrelationships of the data. Consistently high performance measures of the model in terms of ROC-AUC and PR-AUC on simulated datasets demonstrated the ability of scCopulaGNN to discriminate different cell types and maintain an appropriate balance between precision and recall, which is very important for good classification. Moreover, scCopulaGNN also mitigated various forms of variability in scRNA-seq data, including data imbalance. This demonstrates the capability of scCopulaGNN in handling real-world scRNA-seq challenges.

On accuracy against ROC/PR AUC. Accuracy collapses a single threshold and can become better even when PR AUC is getting worse if errors move across classes. ROC AUC, due to prevalence insensitivity, can be good even with heavy imbalance. PR AUC gives the most accurate impression of utility for sparse cell types, which drives our PR centered analysis and stratifying by label size.

### 4.2 Performance between simulated and real-world data

The performance of scCopulaGNN on simulated datasets is effective, demonstrating its capability to accurately classify cell types. However, when applied to real-world data, the advantage is not as pronounced. Overall, across various real-world datasets, scCopulaGNN maintains strong performance, reflected in high scores across different evaluation metrics. This demonstrates scCopulaGNN’s suitability for the scRNA-seq classification challenge. Although in some datasets, compared with baselines, scCopulaGNN's performance is exceptional, its cell-type classification capability remains evident. There are several potential reasons for the varied performance of scCopulaGNN in real-world datasets. A significant factor contributing to this issue is the data imbalance prevalent in real-world datasets which often exhibit significant class imbalances, especially with scRNA-seq data, where certain cell types are underrepresented. Second, the quality and preprocessing of real-world data can vary widely, introducing noise and batch effects absent in simulated data. Moreover, real biological data is considerably more complex and diverse than simulated data such as varying experimental conditions, technical artifacts, and biological diversity. These factors can compromise the model’s ability to accurately identify the correct label.

### 4.3 Key strengths of scCopulaGNN

Interpretability and why copula assists only in conjunction. The ablation indicates that the-only-copula-model is inferior while the-auxiliary-copula-based-combination is competitive or better. This is the pattern we can expect at a first-principle level. A copula with non-informative marginals is not able to discriminate classes—it can just re weight cell configurations together—thus fare poorly in solo-stand-alone mode. When combined with a good marginal predictor, the copula is a structured approach, promoting predictions which are consistent with the learned inter cell relationships at a world wide level; this is particularly beneficial for sparse labels with poor local evidence.

What the copula learns. The precision matrix in the Gaussian copula bivariates conditional dependencies between cells (given features). We see that high-magnitude entries are localized within known cell type neighborhoods in the KNN graph, and large copula contribution edges are consistent with marker gene co expression modules. This fills in attention weights or variational latents by providing a probabilistic, explicitly parameterized dependence term.

Interpretability analysis. We supply three post hoc diagnostics: (i) Node-wise attributions (Integrated Gradients) on the GNN marginal to order genes that are moving each label; (ii) Edge ablations deleting high weight copula edges and printing Δlog likelihood and ΔPR AUC; and (iii) Precision matrix visualization, contrasting copula-derived neighborhoods with known marker-defined ones. Individually, they reveal that the copula tightens up decisions where localized signs are uncertain while preserving confident ones to the GNN. Stepwise procedures and illustrations are in Supplementary S4 (Fig. S10–S12).

### 4.4 Implications for real-world applications

Even though a performance gap between simulation and real-world data exists, the capabilities shown by scCopulaGNN are indicative of large potential of practical applicability. The structure of scCopulaGNN, further refined and tuned, can cope well with the complexities and vagaries of real data, making it more effective overall. The high level of complexity involved, which consequently allows the model to adapt in handling data at different levels of complexity within the simulated datasets, is versatile. This could be directed toward better performances of the model on datasets from the real world through deliberate changes and improvements. Parameter tuning of the model and advanced data preprocessing techniques will, on the other hand, make scCopulaGNN optimal for wider applicability. Lastly, the strengths of scCopulaGNN underlie its value as an indispensable tool in biology research. It is important for a variety of applications ranging from cell-type classification to gene expression analysis and other genomic studies because it is capable of processing highly dimensional data and capturing intricate dependencies.

Our scCopulaGNN represents a significant advancement beyond existing CopulaGNN frameworks by introducing domain-specific adaptations for single-cell RNA sequencing analysis. Unlike previous implementations, we developed a specialized graph construction methodology tailored to cellular transcriptional networks, incorporating unique features such as high-variance gene selection, Poisson-based marginal density estimation, and a refined cell-cell similarity metric. The proposed approach distinguishes itself through targeted architectural modifications: (1) redesigned task-specific layers optimized for binary cell-type classification, (2) enhanced copula parameter estimation techniques that capture complex cellular dependencies, and (3) a robust cross-validation strategy addressing the inherent heterogeneity of single-cell data. Empirical validation across diverse datasets demonstrates substantial performance improvements, with accuracy gains of up to 35 % and ROC-AUC enhancements of 0.2–0.4 compared to the original CopulaGNN framework. These innovations transform the generic graph neural network approach into a specialized, high-precision tool for single-cell transcriptomic analysis, addressing critical limitations in existing methodologies and providing a more nuanced computational framework for understanding cellular heterogeneity.

### 4.5 Potential improvement

Beyond addressing data imbalance issues, several additional enhancements could further augment the performance of scCopulaGNN. First, the quality of input data can be further improved by performing more complex data preprocessing, including batch effect correction and noise reduction. Normalization methods will also improve to reach a unified gene expression level in different datasets. Second, increasing the number of datasets from biological sources and increasing their variety, for example, of different tissues, species, and/or experimental conditions, will actually enable generalization to be much better with increased quantity and diversity in training data. This may also concern datasets on different tissues, species, and experimental conditions that would grow the robustness of the base on which training is performed. Moreover, although currently scCopulaGNN uses GCN, it is possible to think of other models, such as GAT and GraphSAGE, which could dramatically improve the correctness of cell typing.

## 5 Conclusion

In conclusion, scCopulaGNN demonstrates quite good performance on simulated data, particularly under complex dependency and high-dimensional data conditions. These all support its potential effectiveness for the classification of cell types within scRNA-seq data. Thus, in this way, copula theory and graph convolutional network are integrated into a framework that predicts deep relationships inside the scRNA-seq data. In this sense, although performance is relatively gaped compared to real-world datasets, the adaptability of scCopulaGNN and the associated capacity clearly point to great promise for its further development and application. By incorporating refining preprocessing methods, expanding training data, and enhancing model architecture in a way against data imbalances, scCopulaGNN can be optimized further to improve its performance in both simulated and real-world applications.

## CRediT authorship contribution statement

**Shijie Min:** Writing – review & editing, Writing – original draft, Visualization, Validation, Software, Resources, Methodology, Investigation, Formal analysis, Data curation, Conceptualization. **Leann Lac:** Writing – review & editing, Methodology, Investigation, Conceptualization. **Pingzhao Hu:** Writing – review & editing, Supervision, Resources, Project administration, Methodology, Investigation, Funding acquisition, Conceptualization.

## Funding

This work was supported in part by the Canada Research Chairs Tier II Program (CRC-2021-00482), the 10.13039/501100000038Natural Sciences and Engineering Research Council of Canada (RGPIN-2021-04072) and the Canadian Foundation for Innovation (CFI) John R. Evans Leaders Fund (JELF) program (#43481).

## Declaration of Competing Interest

The authors declare that there are no conflicts of interest associated with this manuscript.

## Data Availability

The source code for the sequence vectorization and classification methods used in this study are available in our GitHub repository: https://github.com/shijiemin/scCopulaGNN.

## Footnotes

Appendix A Supplementary data associated with this article can be found in the online version at doi:10.1016/j.csbj.2026.01.010.
