# Supplementary material for: A copula-infused graph neural network for cell type classification in single-cell RNA sequencing data
Source: Comput Struct Biotechnol J. 2026 Feb 2;31:485–99. doi: 10.1016/j.csbj.2026.01.010 (PMC12914865; doi:10.1016/j.csbj.2026.01.010)
Supplement: Figures S1 — Supplementary material [file mmc1.docx]

**A copula-infused graph neural network for cell type classification in single-cell RNA sequencing data**

**Shijie Min**${}^{1*}$**, Leann Lac**${}^{2,3*}$**, Pingzhao Hu**${}^{1,2,4,5,\#}$

${}^{1}$Dalla Lana School of Public Health, University of Toronto, Toronto, Ontario, Canada
${}^{2}$Department of Computer Science, University of Manitoba, Winnipeg, Manitoba, Canada
${}^{3}$Department of Statistics, University of Manitoba, Winnipeg, Manitoba, Canada
${}^{4}$ Department of Computer Science, Western University, London, Ontario, Canada
${}^{5}$Department of Biochemistry, Western University, London, Ontario, Canada
$*$Equal contribution

In this supplementary, we provide the details regarding the performance of proposed scCopulaGNN and baselines in terms of classifying label 1 on Baron3 data, Human Kidney data, mid-sized simulated data, and same-sized simulated data.

### Section 1. Performance of scCopulaGNN and baselines on Baron3 dataset (Label 1)

In this section, we explore the performance of various models on the Baron3 dataset for label 1. The models evaluated include GAT, GCN, MLP, SAGE, SingleCellNet, ACTINN, and our proposed model, scCopulaGNN. As we can see from **Figures S1, S2, S3, and S4**, SAGE achieved the highest accuracy at 0.9472, followed by GCN with an accuracy of 0.9361. The other methods also fared well, with accuracies of 0.8750 and 0.9003 for MLP and ACTINN, respectively. On its part, scCopulaGNN achieved an accuracy of 0.6250, and the method is good at classification in cell types but still has hitches in optimizing for the dataset at hand. As can be seen from Figures 5 and 6, the AUC value for most models approaches 1.0, which means fine performance in distinguishing the cell types under consideration. The AUC for the scCopulaGNN model is 0.9996, just a little less than perfect and therefore remains highly discriminative. In compliance with the AUC results, the PR-AUC values yet again are close to 1.0 in all models, meaning effective approaches for handling class imbalances within the dataset. The model scCopulaGNN has an associated PRAUC of 0.9988, proving that high precision and recall remain constant, while the performance varies with low global accuracy.


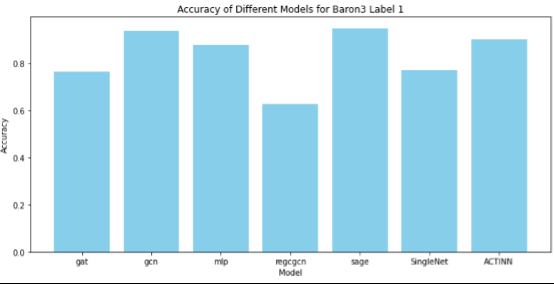


**Figure S1:** Accuracy for Baron3, Label1


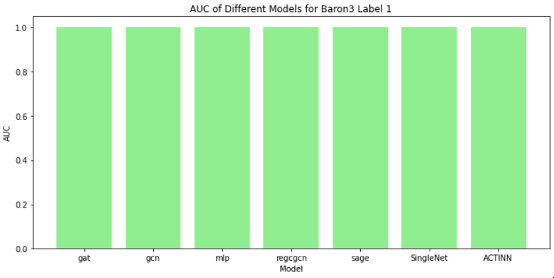


**Figure S2:** ROC-AUC for Baron3, Label1


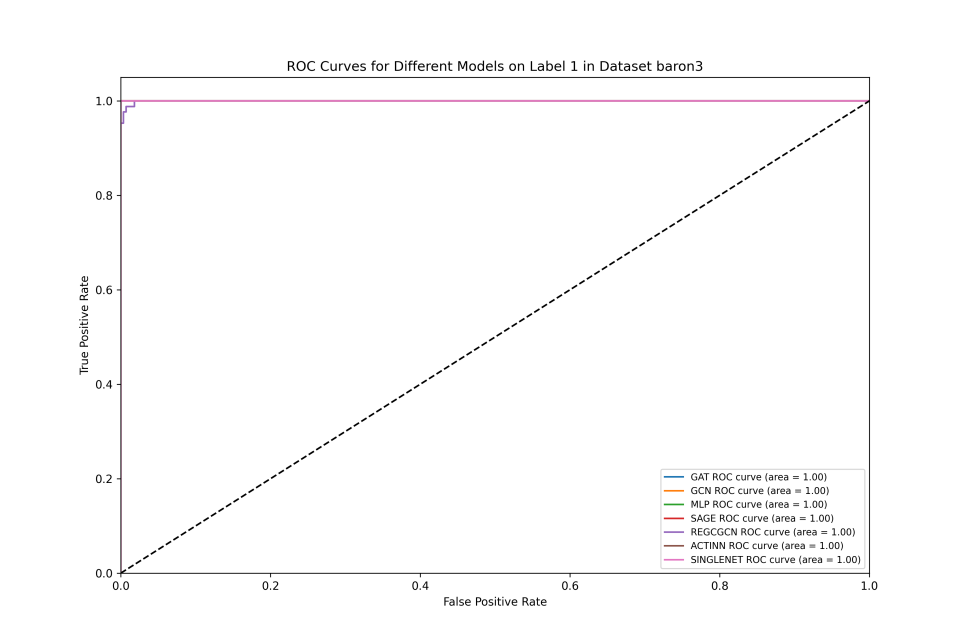


**Figure S3:** ROC Curve for Baron3, Label 1


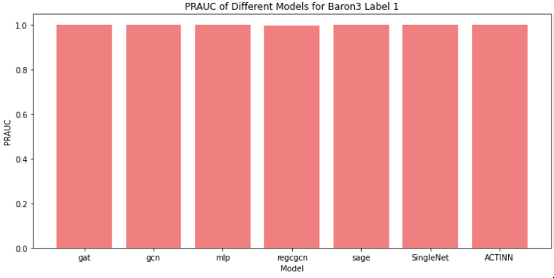


**Figure S4**: PR-AUC for Baron3, Label 1

The results from this experiment have served to show both the strength and the weakness of the scCopulaGNN model: even though it was not the model that produced the best predictive outcome, the model has shown outstanding ROC-AUC and PR-AUC. It means that the model is excellent in distinguishing cell types at maximum precision and recall. This proves that scCopulaGNN captures complex dependencies in graph data particularly effectively by integrating copula theory and graph convolutional networks. However, its lower accuracy indicates that there is room for improvement in either the parameter settings of the model or the training process.

### Section 2. Performance of scCopulaGNN and baselines on Human Kidney Dataset (Label 1)


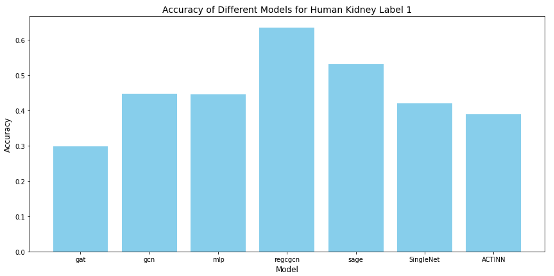


**Figure S5**: Accuracy for Human Kidney, Label 1


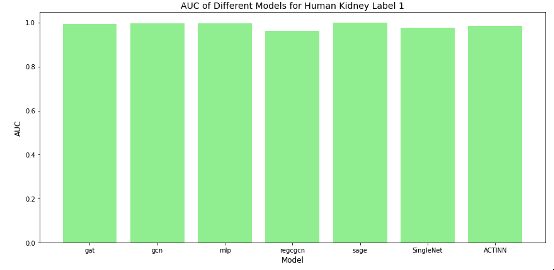


**Figure S6:** ROC-AUC for Human Kidney, Label 1


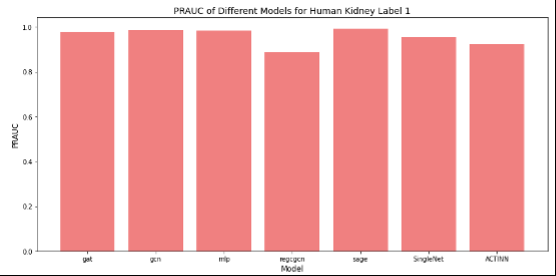


**Figure S7:** PR-AUC for Human Kidney, Label 1

The performance of the scCopulaGNN model on the Human Kidney dataset for label 1 highlights its notable strengths (**Figures S5, S6, and S7**). This model has the highest of all models: 0.6344. In this way, it further demonstrates the effectiveness of scCopulaGNN in enabling precise classification of different cell types within the dataset. In addition, it reached an AUC of 0.9624; hence, it is highly effective for distinguishing among different cell types. Finally, it reached a PR-AUC of 0.8863, confirming that scCopulaGNN can balance both precision and recall well for efficient performance, dealing with the heterogeneous nature of the different cell types represented in the dataset. In general, the performance of the scCopulaGNN model, tested on Human Kidney dataset with labels 1, reiterated its power as a tool for cell-type classification in scRNA-seq data.

### Section 3. Performance of scCopulaGNN and baselines on simulated dataset Z-mid (Label 1)


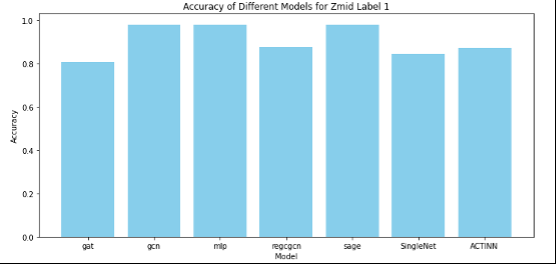


**Figure S8:** Accuracy for Mid-size simulated data, Label 1


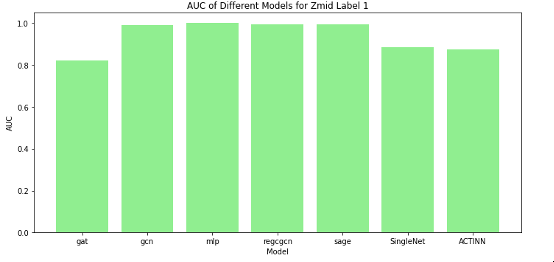


**Figure S9:** ROC-AUC for Mid-size simulated data, Label 1


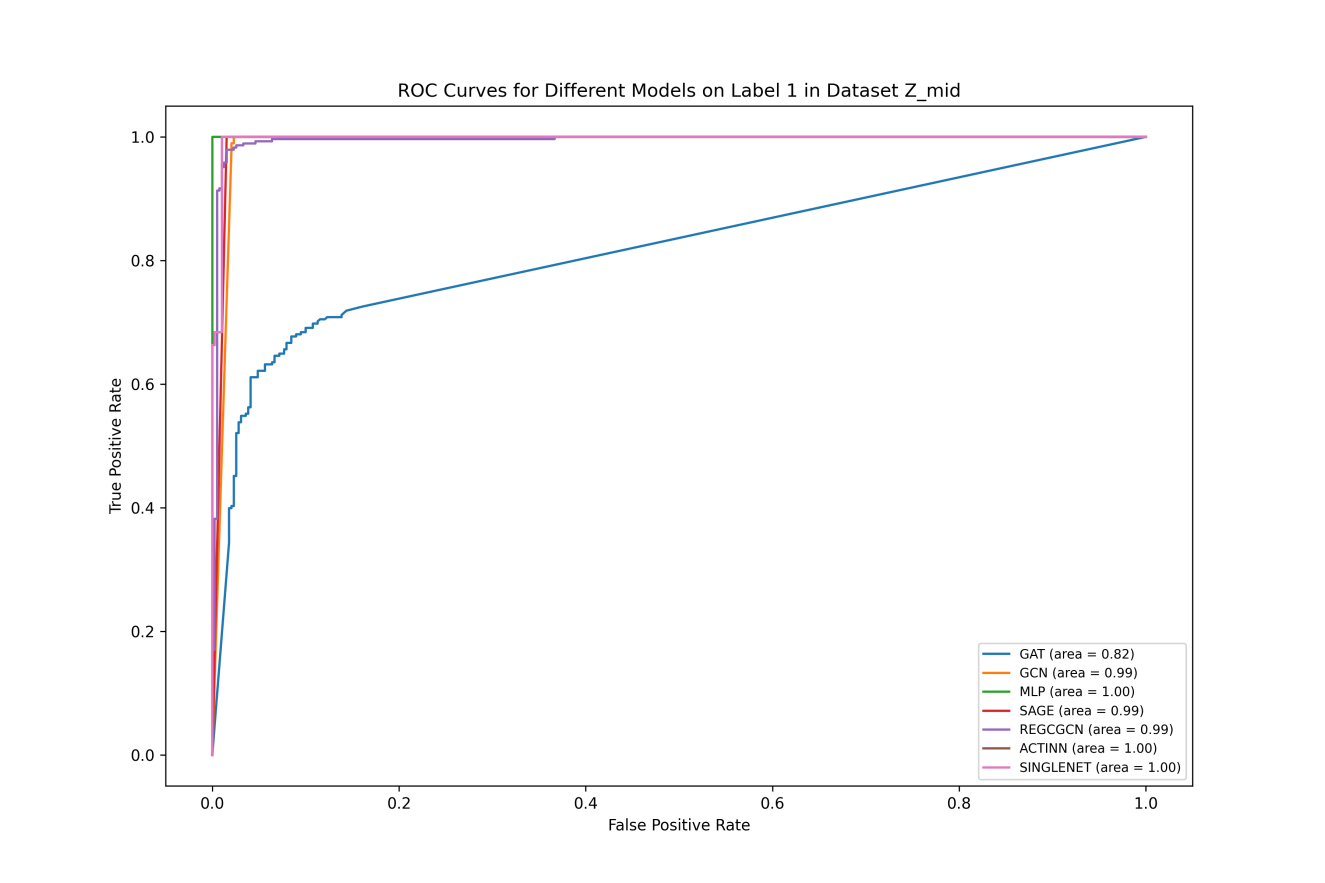


**Figure S10:** ROC curve for Mid-size simulated data, Label 1


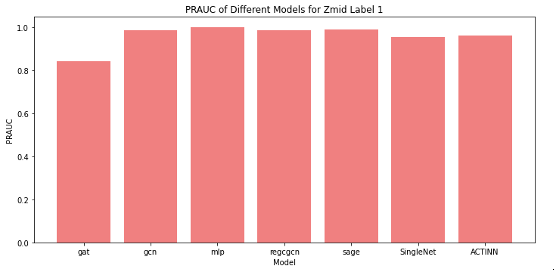


**Figure S11**: PR-AUC for Mid-size simulated data, Label 1

Here, we discuss different labels and how scCopulaGNN performs (**Figures S8, S9, S10, S11**). Label 1, which has the largest number of cells at 2880, shows scCopulaGNN achieving the highest accuracy score, demonstrating its capability in correctly classifying labels. **Figure** **S9** further demonstrates scCopulaGNN’s excellent discriminative ability, as it consistently achieves one of the highest scores among the models. Similarly, in **Figures S10** and **S11**, scCopulaGNN continues to perform well. Compared to other models such as GAT, GCN, MLP, SAGE, SingleCellNet, and ACTINN, scCopulaGNN not only excels in accuracy but also in maintaining high ROC-AUC and PR-AUC values. This consistency across all evaluation metrics highlights scCopulaGNN’s effectiveness in handling the cell-type classification challenge in simulated data.

### Section 4. Performance of scCopulaGNN and baselines on simulated dataset Z-same (Label 1)


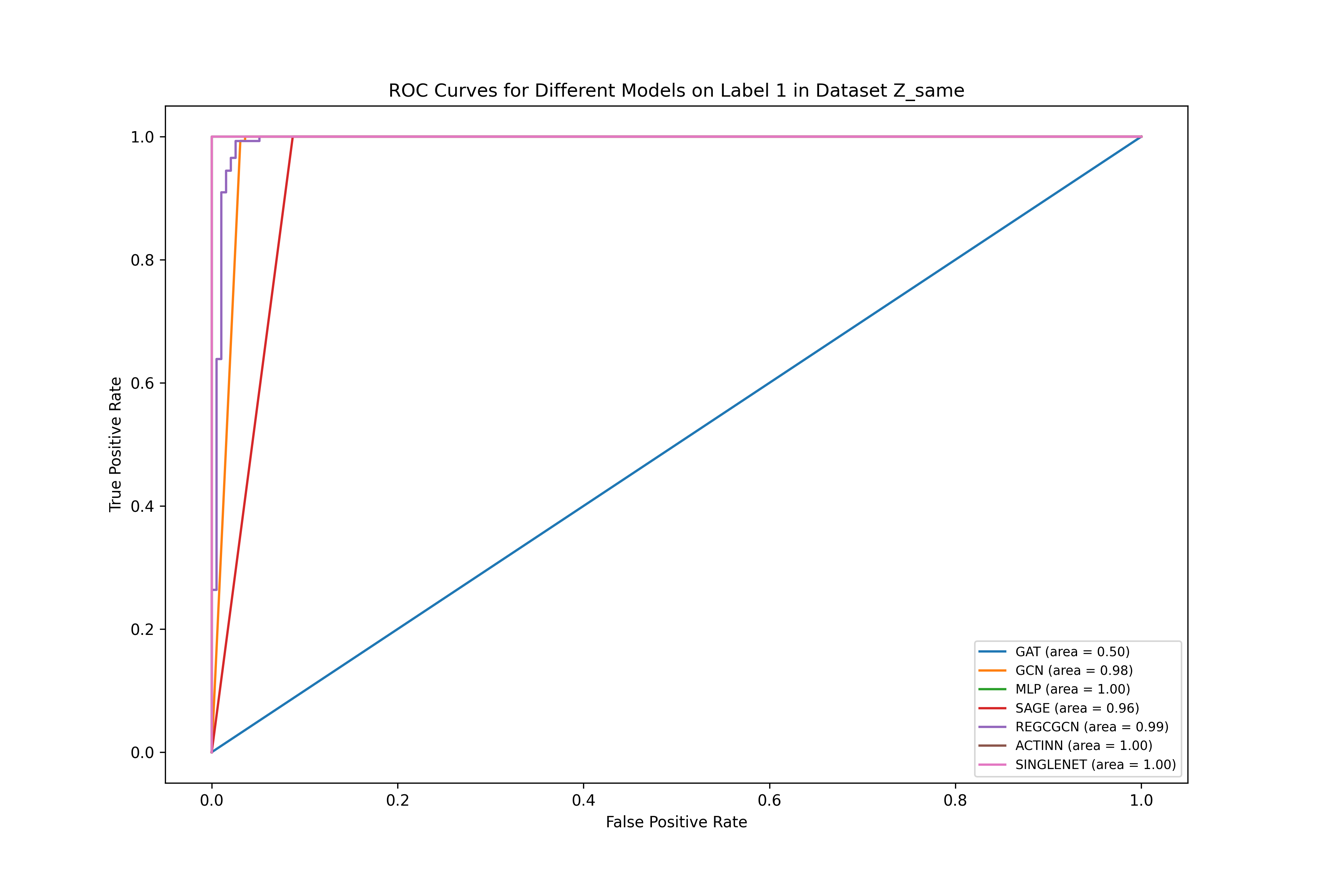


**Figure S12:** ROC Curve for Z_same dataset, Label 1

The ROC curve graph in **Figure** **S12** evaluates the performance of various models on Label 1 in dataset Z_same, which contains the largest number of cells. GAT exhibits an AUC of 0.50, which implies that its performance is no better than random guessing. This could indicate that GAT does not effectively capture Label 1 in this dataset. High AUC of 0.98 in the GCN model supports it having a high ability for good label distinction. The MLP and ACTINN both obtained a full AUC of 1.00, indicating that these models perfectly differentiated between the classes without misclassification either way. Similarly, the SingleCellNet model scored an AUC of 1.00. Besides, the ROC-AUC of the SAGE model is 0.96, and that of the scCopulaGNN model is 0.99. Both show quite high results, which are only slightly lower than the ideal-classifier ones.

In summary, scCopulaGNN delivered the best overall performance with high accuracy, good class discrimination, and balanced precision-recall metrics on all three simulated datasets. Therefore, these results underline the capability of scCopulaGNN in cell-type classification across diverse datasets.

| Copula type | Average accuracy | Average ROC-AUC | Average PR-AUC |
| --- | --- | --- | --- |
| Gaussian copula | 0.4533 | 0.5000 | 0.5382 |
| t-copula | 0.3999 | 0.4120 | 0.4922 |
| Gumbel copula | 0.3100 | 0.3725 | 0.3892 |

Table S1: different copula performance on Human Kidney dataset

| Model | Average accuracy | Average ROC-AUC | Average PR-AUC |
| --- | --- | --- | --- |
| scCopulaGNN | 0.6186 | 0.9704 | 0.9174 |
| scVI | 0.4166 | 0.9601 | 0.9369 |
| scNym | 0.4788 | 0.9871 | 0.9711 |

Table S1: different scRNA-seq classifiers performance on Human Kidney dataset
